# Pandemics of COVID-19 and racism: how HBCUs are coping

**DOI:** 10.3934/publichealth.2021026

**Published:** 2021-03-31

**Authors:** Komanduri S Murty, Tamara B Payne

**Affiliations:** 1 Professor and Char, Department of Behavioral and Social Sciences, Fort Valley State University, Fort Valley, GA, USA; 2 Associate Professor of Rehabilitation Counseling and Director of the Center for Teaching and Learning, Fort Valley State University, Fort Valley, GA, USA

**Keywords:** pandemics, COVID-19, racism, social justice, vaccine, HBCUs, African American, George Floyd, Breonna Taylor

## Abstract

Historically Black Colleges and Universities (HBCUs) are currently facing unique challenges to deal with parallel pandemics of COVID-19 and Racism, given the population they serve (mostly African American) are at high risk of these unprecedented crises. HBCU leaders are adopting various strategies to respond to both the pandemics in order to protect their stakeholders. This paper addresses various models that HBCUs have adopted or planned to adopt to cope with these pandemics, gleaning the data from various secondary sources and selected first-hand interviews with HBCU administrators.

*HBCUs are mobility drivers. They produce 42% of the country's black engineers, 80% of its black judges and 40% of its African American members of Congress. The country's 107 HBCUs enroll 228,000 primarily black students annually, many from poor households, more than half among the first in their families to attend university.*

*—Sylvia Charles and Byron Dobson*

## 1. Introduction

When the COVID-19 pandemic first hit the nation, many academic institutions including Historically Black Colleges and Universities (HBCUs) had to empty residence halls in mid-March 2020 and move all coursework to an online format. While the presidents of these institutions were still wrestling with reopening plans, riots and protests against racism were triggered from Breonna Taylor's shooting by the Louisville Metro Police Department (LMPD) on March 13, 2020 and, the killing of George Floyd by Minneapolis Police Officers on May 25, 2020—causing, as Congresswoman Barbara Lee of California characterizes, a “pandemic within the pandemic,” which the college and university administrators had to address. In this scenario, Historically Black Colleges and Universities^1^ (HBCUs) have a unique role to play for the following two reasons: (1) the disproportionate rates of COVID-19 infection among African Americans (and other minorities) than the general population as shown by the COVID Racial Data Tracker—that nationally blacks are dying at 2.5 times the rate of their white counterparts [1]–[3]; and (2) the ongoing victimization of African Americans because of increasing racial and social injustice. Not only does the cumulative racial trauma contribute to increased levels of acute and chronic diseases among African Americans, as many public health professionals and psychologists found [4], the contextual protests in the COVID-19 environment result in far greater negative impact on the population that HBCUs serve.

Inspired by the Dillard University President Walter Kimbrough's article “No More Statements” in June 2020 [5], Jarrett Carter, Sr. suggested three things that HBCU presidents should do: (1) write and serve as principal investigator for federal grants to support criminal justice reform, crime prevention and victim advocacy; (2) promote the justice work of faculty, students, staff and alumni; and, (3) make social justice a measurable part of the academic mandate [6]. However, these actions require resources. It is no secret that HBCUs have always struggled with fewer resources since their inception than the PWIs (Predominantly White Institutions). Despite losing money from campus housing, dining, sporting events, and other revenue sources when the campuses were closed due to COVID-19, HBCUs had to invest additional funds to upgrade and expand their technologies to accommodate students and faculty for online instruction—all posing a physical and financial threat to their survival [7],[8]. Additionally, many public HBCUs were hit by further budget cuts before re-opening for Fall 2020. Classes started in blending asynchronous, synchronous, and hybrid forms. Students who chose to attend traditional in-person classes were required to wear masks. Faculty who viewed themselves as high risk were given alternative work arrangements to teach online. Campus sports were cancelled for the entire semester [7]. The partial appearance of students and faculty on HBCU campuses also caused revenues to plummet far below pre-COVID-19 levels. Moreover, HBCU land-grant institutions^2^ that were founded through the 1890 Morrill Act continue to be underfunded compared to PWI land-grant institutions previously founded under the 1862 Morrill Act. The impact of the pandemic is even greater among private HBCUs. For example, Dr. George French Jr., president of Clark Atlanta University assessed that each institution in the consortium of the Atlanta University Center (Clark Atlanta University, Morehouse College and Spelman College) could experience losses over $20 million, though inevitable [3]. HBCUs also pay much higher fees than PWIs to issue their bonds, putting them in a greater financial disadvantage in securing funds to meet their operational needs [10].

This paper addresses various methods that HBCUs have adopted or planned to adopt to cope with the current pandemics of COVID and systemic racism, gleaning the data from various secondary sources and selected first-hand interviews with HBCU administrators.

## 2. Materials and methods

This study utilized the COVD Racial Tracker Data (a collaboration between the COVID-19 Tracking Project at *The Atlantic* and the Boston University Center for Antiracist Research) made available for public use through their website (covidtracking.com) to analyze racial disparities in COVID cases, deaths, and hospitalization in the United States through March 7, 2021. Second, the New York Times' “Tracking the Coronavirus at U.S. Colleges and Universities” for COVID cases at selected HBCUs as of November 25, 2020. Supplemental information was also utilized from other reliable data sources (e.g., Centers for Disease Control and Prevention, U.S. Bureau of Census, etc.), scholarly articles from peer reviewed journals, HBCU websites, and news articles.

Finally, we conducted personal interviews with relevant administrative offices of HBCUs (designated by college/university presidents) as specified in the last column of Table 3 between October 12, 2020 and November 13, 2020 to gather information on teaching models implemented at their institutions in Fall 2020 and methods/strategies (if any) employed to protect their students and employees on campus. The timing for interviews allowed institutions to get their infrastructure ready for necessary protection of students and employees, should they choose to offer any in-person classes on campus and complete interviews before they get ready to close for Thanks Giving holidays. Initially, all HBCUs were aimed for interview, but only those who responded positively to the first three requests (n = 21) were interviewed at a mutually agreed upon time for a telephone interview.

## 3. Results

### 3.1. COVID-19 and vulnerable minorities

COVID-19 infections and deaths have disproportionally affected African Americans and other minorities [12]. As shown in Table 1, the nation as a whole has lost 73,462 (or 14%) Black/African American lives as of March 7, 2021 because of COVID-19; i.e., at the rate of 178 per 100,000 persons—1.4 times to that of whites (124 per 100,000 persons). Their infection rate is 1.3 times and hospitalization rate is 2.2 times to those of whites. Hispanic/Latino persons died at the rate of 154 per 100,000 persons (n = 84,287) or 1.3 times to that whites. Their infection and hospitalization rates are 1.5 and 1.7 times to those of whites. American Indian/Alaska Native as well as Native Hawaiian/Other Pacific Islander persons have also experienced disproportionate number of deaths, infections, and hospitalizations.

These differences are far greater at different times and in different regions. For example, CDC National Vital Statistics System (NVSS) show that Blacks accounted for 18.7% of 114,411 COVID-19 related deaths during May-August 2020 [13]. Regionally, African Americans accounted for 46 percent of COVID-19 infections in Washtenaw County, Michigan, 46 percent of COVID-19, though they only represented 12 percent of the region's population [14]; and, 70 percent of COVID-19 related deaths in Chicago, Illinois, where they represented only 29 percent of population [15]. Likewise, Latinos accounted for more than 20 percent of COVID-19 infections, although they represented only six percent of the population in Iowa; and, 31 percent of COVID-19 cases in Washington State, despite their representation of only 13 percent of the state's population [16]. Killerby et al. [17] found in their study based on 220 hospitalized and 311 non-hospitalized COVID-19 patients from six metropolitan Atlanta hospitals and associated outpatient clinics found that Black patients with COVID-19 were more likely to be hospitalized (79% among hospitalized v. 45% among non-hospitalized) than White patients (13% among hospitalized and 29% among non-hospitalized) apart from other characteristics such as age (65 years or older), having diabetes mellitus, lack of insurance, male sex, smoking, and obesity.

**Table 1.** COVID-19 infection cases, deaths, and hospitalization by race, through March 7, 2021.

		Number	Per 100,000 Population	Cases (%)
Cases	Total	28,849,461	7,588	100.1
	White	10,579,600	4,481	36.7
	Black/African American	2,400,861	5,817	8.3
	Hispanic/Latino	3,724,219	6,804	12.9
	Asian	564,979	3,144	2.0
	American Indian/Alaska Native	223,837	8,153	0.8
	Native Hawaiian/Other Pacific Islander	54,267	9,055	0.2
	Multi-racial	164,730	1,532	0.6
	Other	1,565,314	9,764	5.4
	Unknown	9,602,639		33.3
Deaths	Total	524,683	138	100.0
	White	292,777	124	55.8
	Black/African American	73,462	178	14.0
	Hispanic/Latino	84,287	154	16.1
	Asian	17,071	95	3.3
	American Indian/Alaska Native	4,722	172	0.9
	Native Hawaiian/Other Pacific Islander	863	144	0.2
	Multi-racial	1,936	18	0.4
	Other	15,550	97	3.0
	Unknown	34,155		6.5
Hospitalizations	Total	618,312		100.2
	White	288,383	122	46.6
	Black/African American	110,220	267	17.8
	Hispanic/Latino	114,833	210	18.6
	Asian	20,619	115	3.3
	American Indian/Alaska Native	12,656	461	2.0
	Native Hawaiian/Other Pacific Islander	2,250	375	0.4
	Multi-racial	3,058	28	0.5
	Other	21,933	137	3.5
	Unknown	45,342		7.3

Note: Source: The Atlantic (March 7, 2021) The COVID Racial Data Tracker. Available from: https://covidtracking.com/race.

While the widespread testing and limited reporting^3^ may raise some concerns regarding the accuracy of variations by race and ethnicity, scholars tend to attribute vulnerabilities of minorities during pandemics more frequently to social factors than biological factors. For example, Dorothy Roberts, the author of *Fatal Invention: How Science, Politics, and Big Business Re-create Race in the Twenty-first Century* concluded his preface thus: “After five years of intense research and soul-searching, I found not one shred of evidence to counter my belief in the political nature of race. In fact, my journey only strengthened my understanding of our common humanity and the dehumanizing consequences of believing in innate racial differences. The answer to the problem of race will not be found in our genes. Yes, human beings are remarkably similar at the genetic level. But what should link us together not our genetic unity; we should be bound by a common struggle for the equal dignity of all of humankind” [19]. Yearby and Mohapatra [20] also viewed the racial and ethnic disparities in COVID-19 infections and deaths as direct result of historical and present practices of racism mitigated through disparities in exposure (because many racial and ethnic minorities are employed in “essential jobs” that require them to interact with others), susceptibility (because of racially segregated neighborhoods with severe health-related housing violations^4^), and treatment (because of poverty, lack of access to healthcare, etc.). These social inequities result in health inequities, which create syndemic conditions^5^, which cause unequal risk for COVID-10 among African Americans and other minorities by accelerating their exposure, disease, and mortality [12].

### 3.2. COVID-19 and HBCUs

Nearly 30 million cases—sometimes growing at the rate of more than 200,000 cases per day and a million a week—and about 535,000 deaths in little over a year since the first COVID-19 patient was diagnosed in Washington state on January 20, 2020—is the tragic record of COVID-19 in the United States. As discussed earlier, African Americans (and other minorities) are at the highest risk of dying from the COVID infection [2]. Dr. Irving Pressley McPhail, the 12^th^ president of Saint Augustine's University in Raleigh, North Carolina, died on October 14, 2020 due to the COVID-19 virus and associated complications [23]. While this may be the ultimate price to pay for contracting this deadly virus, many faculty, staff, and students are susceptible to suffer from it unless effective emergency strategies are designed and implemented.

Prevalence of COVID Infection Among College Students and HBCUs: Table 2 shows the number of reported infection cases by colleges and universities and selected HBCUs.

**Table 2.** Tracking COVID cases at selected HBCUs as of November 19, 2020.

HBCUs By State	COVID cases
Alabama (30 colleges and universities)	10,950
Alabama A&M University—Huntsville	41
Alabama State University—Montgomery	2
Tuskegee University—Tuskegee	110
Arkansas (26 colleges and universities)	6,329
University of Arkansas at Pine Bluff—Pine Bluff	184
District of Columbia	617
University of the District of Columbia	3
Howard University	39
Florida (115 colleges and universities)	16,001
Bethune Cookman University—Daytona Beach	47
Edward Waters College—Jacksonville	10
Florida A&M University—Tallahassee	195
Georgia (37 colleges and universities)	12,626
Albany State University—Albany	141
Fort Valley State University—Fort Valley	98
Savannah State University—Savannah	54
Kentucky (53 colleges and universities)	8,314
Kentucky State University—Frankfort	124
Louisiana (38 colleges and universities)	5,783
Grambling State University—Grambling	60
Southern University and A&M College—Baton Rouge	202
Southern University New Orleans—New Orleans	15
Maryland (20 colleges and universities)	3,131
Bowie State University—Bowie	75
Coppin State University—Baltimore	8
University of Maryland—Eastern Shore	40
Morgan State University—Baltimore	17
Mississippi (15 colleges and universities)	3,553
Alcorn State University—Lorman	128
Jackson State University—Jackson	42
Mississippi Valley State University—Itta Bena	112
Rust College—Holly Springs	39
Missouri (37 colleges and universities)	10,504
Harris-Stowe State University—St. Louis	46
Lincoln University—Jefferson City	14
North Carolina (48 colleges and universities)	11,929
Elizabeth City State University—Elizabeth City	75
Fayetteville State University—Fayetteville	140
Johnson C. Smith University—Charlotte	1
North Carolina Central University—Durham	105
North Carolina A&T State University—Greensboro	475
Shaw University—Raleigh	27
Winston-Salem State University—Winston Salem	85
Ohio (61 colleges and universities)	15,283
Central State University—Wilberforce	40
Oklahoma (21 colleges and universities)	5,079
Langston University—Langston	47
South Carolina (29 colleges and universities)	11,464
Benedict College—Columbia	6
Claflin University—Orangeburg	18
South Carolina State University—Orangeburg	41
Tennessee (66 colleges and universities)	9,486
Tennessee State University—Nashville	55
Texas (84 colleges and universities)	26,157
Prairie View A&M University—Prairie View	418
Texas Southern University—Houston	46
Virginia (41 colleges and universities)	8,493
Hampton University—Hampton	12
Norfolk State University—Norfolk	24
Virginia State University—Petersburg	6
Virginia Union University—Richmond	16
West Virginia (19 colleges and universities)	1,785
Bluefield State College—Bluefield	17
West Virginia State University—Institute	71

Note: Source: Compiled from “Tracking the Coronavirus at U.S. Colleges and Universities”, New York Times, November 25, 2020. Available from: https://www.nytimes.com/interactive/2020/us/covid-college-cases-tracker.html.

As noticed in the above table, HBCUs experienced relatively fewer cases than non-HBCUs (as well as among the general population) in each state^6^. This is because: (1) HBCUs managed to attain higher student compliance with the institutional guidelines in terms of social distancing and mask wearing; (2) HBCU leaders came up with practical but effective strategies for employees' return to campus, and methods to monitor, trace, and report COVID cases that all institutional employees and students could adopt without a great deal of difficulty; (3) HBCU instructors adopted alternative instructional strategies to allow students to attend classes either in-person or virtually based on their circumstances; and, (4) HBCU students became more knowledgeable of the COVID risk to themselves, their families and communities through consistent weekly-alerts, which may have resulted in a higher rate of self-protection from the virus. As viewed by Todd Simons, associate vice chancellor for university relations at North Carolina A&T, “the stakes are high” and “they are more ready to police themselves with regard to COVID protection, and that could be an important reason why our campuses are not experiencing big clusters or outbreaks” [25].

Phased Return of Employees to Campus: HBCU leaders, as in the case of Fort Valley State University, included a staggered approach aimed to spread out staff spatially and in the time of their return to campus. This approach allowed bringing employees in three phases before the classes began by categorizing them in groups based on identified work requirements. Phase I was divided into two groups—Phase 1(a) employees (facilities/plant operations staff and staff on non-closure emergency leave) were to return no later than June 1, 2020; and Phase 1(b) employees (administrative/operations staff; campus safety operations; Child Development Center, Head Start and Early Head Start staff; residence life start, and research/laboratories/Farm Animal staff) were to return on June 15, 2020. Likewise, Phase II was divided into two groups—Phase II(a) employees (enrollment management and student affairs staff, and coaches) were to report on July 13, 2020; and Phase II(b) employees (residence assistants and all other staff, faculty on 12-month appointments, and department chairs) were to do so on July 27, 2020. Finally, Phase III included 10-month faculty and employees not included in above categories who were asked to report on August 3, 2020, one week before the start of classes, thus making campus fully operative before the Fall semester started [28].

Alternative Teaching Models: Moreover, the HBCU leaders implemented alternative teaching models appropriate to their campuses in an effort to keep their student populations and other stakeholders safe. Table 3 presents a variety of such models as provided by the institutional administrators. They combine the traditional face-to-face and online formats by integrating available technologies and educational platforms.

Telework, Flextime, and Occasional/Intermittent Extensions: Telework and Flextime provisions were implemented initially when campus shutdowns occurred (except for emergency services) in the mid-Spring semester of 2020 because of the COVID outbreak. However, many HBCU leaders extended these alternative work arrangements along with Occasional/Intermittent work arrangements on the need basis of their eligible staff and faculty members in the following Summer and Fall Semesters to minimize their exposure to virus and risk of getting infected—within the scope of institutional and/or university system policy guidelines.

Shortening/squeezing Semesters: HBCUs like Central State University in Ohio and Fort Valley State University in Georgia, which could not shift completely to online instruction in Fall 2020, shortened/squeezed their semesters by either starting late and/or ending in November before the flu season began [29]. Fort Valley State University also adjusted its academic calendar to end classes on November 21 by moving all holidays including Thanksgiving to the end of the semester. Similar methods were adopted by Lincoln University, Delaware State University, Cheney University, and others [30].

Other Safety Measures: Some colleges also invested in computers and purchasing additional technological equipment to meet students and instructional needs. For example, Edward Waters College in Jacksonville, Florida secured 700 computers for distribution to students to enable them to continue taking courses online [31]; and, Fort Valley State University in Georgia invested $100,000 to upgrade classrooms for blended synchronous instruction. Further, some colleges developed emergency management plans for ongoing testing, tracing, and implementing mandatory masking and social distance measures. For example, Fort Valley State University has created a cabinet level Emergency Management Office, which oversees the adequate supply of personal protection equipment (PPE) such as masks, gloves, face shields, Plexiglas barriers, and hand sanitizers to each building on the campus; and, coordinates sanitizing classrooms and offices, displaying signs on campus, monitoring limited crowds in lobbies and elevators, taking temperatures of everyone entering campus anytime daily, reporting cases infected, and quarantining them and their close associates. Weekly reminders were sent to the entire campus community under the running message “*Our safety is our collective responsibility. Do your part to keep our community safe*.” These weekly reminders suggested protective measures by self-monitoring health on a daily basis as well as during the upcoming Flu season, along with a resource link from CDC.

**Table 3.** Teaching models implemented by HBCUs in Fall 2020.

HBCU	Teaching Model	Information Provided by:
Albany State University	Hybrid, Fully online (alt days), F2F (limited capacity	Academic Affairs
Alcorn State University	Started F2F (limited capacity) changed to grouping using alt days and Friday fully online; Graduate is fully online	A. Provost for Research
University of Arkansas at Pine Bluff	75% online or hybrid; 25% F2F using Blackboard, Zoom and Collaborate	Assistant Vice Chancellor of Academic Affairs
Bennett College	Fully Online	Academic Affairs
Bethune-Cookman	½ F2F (limited capacity) and ½ online Hybrid; Online synchronous use recorded lectures; ORE for non-majors, sciences, Freshmen and Sophomore; use Canvas	Academic Affairs
Central State University	Small F2F classes; Remote companion sections changed to synchronous; Hybrid using alternate days	Academic Affairs
Coppin State University	Fully Online; 10% F2F (limited capacity) for the majority of sciences; COVID-19 courses- online or synchronous with alt days	Registrar's
Delaware State University	Majority Fully online use Blackboard; Select course are F2F such as aviation, clinics and nursing: Classes are small sizes	Academic Affairs
Fisk University	Started as hybrid and went to fully online	Academic Affairs
Fort Valley State University	Fully online, hybrid, and blended synchronous (voluntary selection for students to be F2F or remote)	Academic Affairs
Grambling State University	Students registered for classes and classrooms were set up for capacity based on the number of registered students. F2F uses Canvas and Zoom	Academic Affairs
Hampton University	Fully online	Chancellor & Provost
Howard University	Fully online	Academic Affairs
Kentucky State University	Hybrid with alternate days; Fully online; Labs and clinicals are F2F	Academic Affairs
Lincoln University	F2F with alternate days (limited capacity); Hybrid using Canvas	Provost
Morehouse College	Fully online	Academic Affairs
Morris Brown College	Fully online	Academic Affairs
Savannah State University	Fully online; Hybrid	Academic Affairs
Spelman College	Fully online	Academic Affairs
Tennessee State University	Started as hybrid changed right before school, but students were initially registered to fully online	Academic Affairs
Tuskegee University	Started as hybrid and transitioned to fully online	Academic Affairs

Note: Source: Personal interviews, October-November 2020. F2F = Face-to-Face; ORE = Oxford Research Encyclopedia program.

Fort Valley State University also established a no-cost to students COVID-19 surveillance testing program as an integral part of the university's multi-pronged strategy to mitigate and monitor the spread of COVID. This program tests random samples of students for COVID regardless of whether they have a known exposure or are showing symptoms of COVID. This allowed the university to make inferences about the level of spread in the student population and identify asymptomatic cases for isolation. The university has been testing a randomly assigned sample of 5% of students on each floor of the residence halls each week, initially by using a Diagnostic Saliva Test and later on supplementing it with the Rapid Antigen Test, which allows a quick result within 15 minutes. Students selected for testing are notified via their university email to schedule a time for the test, along with detailed scheduling instructions and information for taking the test. Randomly selected students were excluded for testing in subsequent weeks. Students were made aware that failure to respond and schedule a test may result in a directive to quarantine until testing is completed, loss of access to university spaces, and a report to Student Conduct [32]. As Robin Featherstone, Shaw University Director of Student Activities, Leadership and Greek Life said, “our community is vulnerable and some students here know that they are in a safe place—in a (private) room where they can get up and go eat when they need to” [1].

Preparing for future adversities: Dr. Paul Jones, the President of Fort Valley State University, saw a need to prepare the university for any adverse events, not just for COVID-19, in coming years. Therefore, he put in place a Business Continuity Plan (BCP) and a team of members (composed of faculty, administrators, and staff) to implement the plan as an ongoing strategy, in October 2020. The primary objectives of the BCP are to: (1) minimize loss to the university; (2) ensure continuity of instruction and quality services to students and other institutional constituents; (3) resume administrative operations efficiently; (4) facilitate awareness and training, accountability, data accuracy and resilience, and financial assurances; (5) conduct a risk analysis of the mission-critical functions of each of the administrative units on campus, combined with the strategies and responses required to resume and/or recover stable and secure business functionality in the event of significant disruption of services; and (6) provide guidelines for a more sustained, university-wide, disaster response, among others. The plan is sought to be implemented by the BCP Team and several Unit Command Teams, who are responsible for providing the leadership and coordination required to maintain or recover operations. The president or his designee is ultimately responsible for directing or coordinating the implementation and annual review of the BCP. The implementation of the initial BCP involves the following phases: (1) BCP Testing; (2) Pre-planning Phase-1; (3) Pre-planning Phase-2; (4) Work Plan, Processes and Reporting: Phase-1; (5) Work Plan, Processes and Reporting: Phase-2; and, (6) Review and Modification for Next Year implementation. This strategy of BCP combined with the Emergency Management Plan (EMP) is thought to increase the resilience of the institution in case of unforeseen disastrous events, whether they are acts of nature (e.g., tornadoes, hurricanes, etc.), human-made disasters (e.g., acts of terrorism), health crises (e.g., COVID-19, Ebola, etc.) or technological failures [33].

### 3.3. Racism and HBCUs

While keeping campuses safe from COVID-19, HBCUs have been thrust into the spotlight due to social unrest in the form of riots and protests across the nation against racism and police brutality that caused the deaths of George Floyd and Breonna Taylor, among others. Like COVID-19, racism is also a public health crisis. Many states like Michigan, Nevada, Pennsylvania, Wisconsin, along with local governments in California and Kentucky have already declared racism a public health crisis [34],[35]. In June 2020, California Democratic Congresswoman Barbara Lee introduced legislation (HR 100) to create a federal Commission on Truth, Racial Healing, and Transformation (TRHT) to examine the effects of slavery, institutional racism, and discrimination against people of color, and how the nation's history impacts its laws and policies today. She said that Black Americans, who are disproportionately affected not only by COVID-19 crisis but also by high levels of police violence, are experiencing “a pandemic on top of a pandemic,” and the underlying root cause of both is generations of systemic racism [36]–[38]. On the contrary, Larry Hogan, Maryland's Republican Governor vetoed a racial discrimination settlement that would have provided $580 million to four HBCUs in the state—the money that would have helped those institutions significantly [39].

In a virtual town hall symposium on COVID-19, systemic racism, and the responses of HBCUs, Dr. Reynold Verret, president of Xavier University of Louisiana said that, *“we are in a moment of truth telling...in a very graphic way of what our history is about... Young people...of all races are grasping that notion that they were not told the truth. We are one of the important grounds to have those difficult discussions that need to be had. The value of HBCUs is becoming seen as a place where Americans come to learn from us, not the other way around”* and Dr. Mary Schmidt Campbell, president of Spelman College of Atlanta echoed, *“I see HBCUs having this deep investment in our own history, which is the history of our country and the truth of our country, which I think can help our other young people come to grips with what we really need to do in order to fully heal”* [40]. Dr. Aaron Walton, President of Cheney University and Dr. Brenda Allen, President of Lincoln University issued a joint statement denouncing the unconscionable killings of George Floyd, Breonna Taylor, and Ahmed Arbery that have drawn national and international attention and calling on students to *“focus their energy on bringing constructive change to the society ... use the modern technology and resources ... to overcome inequalities and to change our communities for the better... express their voices at home or in person at peaceful demonstrations”* [41]. In her statement, Dr. Ruth J. Simmons, President of Prairie View A&M University, laid out some action plans: (1) Cease equivocation about the root causes of the current social upheaval that divides the country by enabling students to “understand the impact of the racialized history of the United States on their personal as well as professional lives”; (2) acknowledge courageous members of the community who have advocated and worked on behalf of justice for all peoples by creating Activist in Residence position, and establishing a Sandra Bland/Robbie Tolan Award to annually recognize those who have increased understanding in the area of criminal justice reform; (3) ongoing engagement in the persistent issue of racism and discrimination to highlight the role that the University is obligated to play; and (4) establish a Center for Race and Justice to encourage teaching and scholarship that “positively contributes to overturning systemic biases that impede the ability of minorities and other groups to be accorded their full rights under the U.S. Constitution” [42]. Other HBCUs also responded by creating new centers for Racial Justice. For example, Shaw University announced the creation of its Center for Racial and Social Justice to enable meaningful social change by fostering engagement around civil and human rights, and social justice; the University of the District of Columbia launched the Institute for the Study and Elimination of White Supremacy in America; and Dillard University in New Orleans is starting its Center for Racial Justice [43]. Fort Valley State University is also contemplating on launching a Center for Social Justice, but in the meantime, it is initiating a concentration in social justice within the curriculum of its Organizational Leadership Program.

### 3.4. Continued challenges

Despite many foregoing efforts of HBCU leaders in protecting their campuses and constituents from COVID as well as taking necessary steps to provide a much-needed response to ongoing systemic racism in the nation, they continue to face some major, but inevitable, challenges:

1. Financial Challenges—Enrollment fluctuations, scholarships and living expenses to needy students, increased student services, cancelled athletic activities, reduced dorm occupancies, technological infrastructure needs, faculty and staff trainings, etc. continue to pose financial challenges to many HBCUs [44], which were operating with bare minimum budgets even before the COVID outbreak due to severely steep declines in federal funding between 2003–2015 [45] and facing budget cuts in the current academic year [46]. It is true that the United Negro College Fund, Thurgood Marshall College Fund, and some corporations tried to help financially strapped HBCUs; and, the federal government provided stimulus funds through the CARES Act and FUTURE Act to relax immediate strain on these institutions [25]. However, HBCUs don't get the philanthropic funds from rich donors like PWIs and many of them don't have large endowments, yet their financial needs are far greater and their long-term survival remains a challenge. Even HBCUs like Morehouse College (with a relatively sizable endowment compared to many HBCUs) had to take drastic steps like furloughing 54 part-time and full-time employees for two months effective June 1, 2020; 10–15% pay cuts to faculty and staff with salaries more than $55,000 and a 25% pay reduction to President David Thomas himself for the academic year [47].

2. Accreditation—Some HBCUs are stalled in the middle of their preparation for accreditation affirmation. Administrators of these institutions have to continue their efforts to complete the process by coordinating various offices and financial audits with employees utilizing telework/flextime provisions and those struggling between doctors' appointments and work schedules, while safeguarding their own health. In a recent webcast hosted by Prairie View A&M University, Dr. Belle S. Wheelan, president of Southern Association of Colleges and Schools Commission on Colleges (SACSCOC) made clear that, *“because every institution is impacted by this [COVID-19], the peers that will come to evaluate compliance understand because they are having to go through the same thing. However, it does not relieve anybody of meeting the standards”* [48]. Even before COVID-19, a report from the Fredrick D. Patterson Research Institute of the United Negro College Fund [49] observed that four UNCF institutions lost accreditation since 2000 and nine of its 37 institutions were sanctioned between 2015 and 2018. The study recommended that SACSCOC should: (1) create clear guidelines for peer definitions, training to reduce implicit bias and peer assignments that take institutional time and mission into consideration; (2) have an independent third party complete an expert review of its processes, standards and policies investigating the presence of implicit bias against certain types of institutions; (3) sanction that data be publicly available to allow for greater transparency about sanction decisions; and, (4) establish clear guidelines for how institutions should present their financial stability based on institutional type and resources.

3. Mental Health: HBCUs continue to observe widespread anxieties among their constituents' online chats every time they learn about the death of a faculty/staff member or student or a loved one. Some worry about lack of access to Wi-Fi, laptops, and/or cellphone data to complete assignments. To some, online instruction, which they never had before, could trigger anxiety around possibly failing the course or receiving a lower grade. Others develop anxiety because of isolation or inability to go to work or loss of employment. Historically, African Americans are known to underutilize mental health counseling and treatment services as observed by the National Institute of Health and Black Mental Health Alliance [50], and now that those services are only virtually available in most cases, the chances of utilizing them by many African American students is even more slim. Therefore, HBCU leaders face a challenge to devise ways of providing emotional emancipation circles, reaching their needy population via virtual town hall meetings, motivating them to take therapy sessions available to them, and funding those resources.

4. Vaccination: While Pfizer and Moderna COVID-19 vaccines have just been approved for emergency use and AstraZeneca is yet to be approved by the FDA, CDC's Advisory Committee on Immunization Practices (ACIP) identified four groups for receiving them first: healthcare personnel, other essential workers for continuing critical infrastructure, adults with underlying health conditions such as diabetes and heart disease, and adults over the age of 65 years [51]. However, the concern remains about its effectiveness for African Americans^7^, which require their participation in clinical trials. Leading the way, two HBCU presidents—Dr. Walter M. Kimbrough of Dillard University and C. Reynold Verret of Xavier University—participated in a vaccine trial for COVID-19 and encouraged students to do the same. Verret said, *“It is important to have people like us in these trials. ... We only have 1 or 2 percent; we need 10 to 15 percent participation”* [55]. Whether the trials reach the needed representation remains to be seen.

## 4. Conclusions

Despite decades of underfunding, HBCUs have struggled through and survived many crises throughout their existence and positioned themselves as the nation's most vital and vibrant institutions in U.S. higher education [9]. However, it is not without sacrifices, resilience and hard work of many people who lead these institutions. Now they are once again struck with two pandemics—one being the worst health crisis in 100 years (COVID-19) and the other being systemic racism, notably the recent events surrounding the murder of George Floyd and other innocent African Americans, which is rooted in centuries of anti-Black racism. The only difference is that the current pandemic of COVID-19 is affecting entire world, though the United States has most cases and HBCUs in the nation are the most vulnerable. HBCU leaders have been doing all they can to respond to both pandemics as discussed in this paper. Simultaneously, they are also adapting to the serious financial challenges that have begun to unfold and are likely to continue for at least some time. To tackle them, HBCUs are gearing up to work collectively to achieve their common goals by forming coalitions and partnerships not only among HBCUs themselves but also with governments and corporations. For example, Howard University formed the HBCU Emergency Management Consortium (HBCU-EMC) and leaders of 49 HBCUs responded to the consortium's survey in March 2020—a collective effort to build the capacity of HBCUs as academic and community resources for emergency management and homeland security. As Downer pointed out *“HBCUs with their decades of experience promoting the health and wellbeing of marginalized communities are well positioned to lead discussions on culturally responsive disaster prevention and relief in the time of natural disasters and/or public health crises”* [56]. Further, the strength of partnering with corporations can be seen from Netflix Corporation's scholarship donations of $40 million each to Morehouse and Spelman colleges [57]. And, because of the concerns raised by HBCU presidents, the federal spending bill increased funding to HBCUs by $35 million, making it a total of $280 million for 2018 [58]; and in 2020, the FUTURE ACT, while making permanent $255 million in annual STEM funding for the minority-serving colleges, specifically allocated $85 million to HBCUs [59]. As we continue to move forward with the ongoing efforts on the part of HBCU leaders, the expanded distribution of COVID-19 vaccines, and the unprecedented choice of President-elect Joe Biden to form the government with men and women from diverse racial/ethnic backgrounds, we can be optimistic about HBCUs finding relief from COVID-19 and helping heal the nation from polarized racial/ethnic tensions.

## Footnotes

1 Historically Black Colleges and Universities (HBCUs) are black academic institutions of higher learning established prior to 1964 with principal mission to educate black Americans. Historically, they have served a population that has lived under severe legal, educational, economic, political, and social restrictions. The composition and position of the black population have influenced the development of HBCUs, and in turn, HBCUs have contributed much to the advancement of the black population. Traditionally, in comparison with other colleges, HBCUs are poor in terms of financial resources, physical plant, and teaching facilities; they have faced opposition from the white power structure; and, they have dealt with many students who are not adequately prepared for higher education. They are a vital national resource and have served as the fulcrum of African-American Leadership. Currently, there are 101 HBCUs (51 public and 50 private non-profit). Twenty-seven HBCUs offer doctoral programs, 52 offer master's, 83 offer bachelor's, and 38 offer associate degree programs [9].

2 Seventeen public HBCUs now in existence were established under the second Morrill Act of August 30, 1890, which paved the way for the development of legally separated black and white public colleges in border and southern states. During the period from 1890 to 1899, one black college was either planned or founded per year in each of the seventeen border and southern states. They were unequal and did not offer four-year college programs. The legacy of the industrial, mechanical, and agricultural education of blacks in the South stems from this period, and all of the schools established under the Morrill Act offered degrees later on. Actually, the public HBCUs were created by the southern state governments for three reasons: (1) to get millions of dollars in federal funds for the development of white land-grant universities; (2) to limit black education to vocational training; and (3) to prevent blacks from attending white land-grant colleges. Following Reconstruction (after 1877), southern legislatures enacted a host of segregation laws, including those that excluded blacks from all the white educational institutions. Yet the region had received federal funds for designated white institutions since the passage of the first Morrill Act in 1862. In order to prevent continued discrimination against blacks in public higher education, the federal government enacted the second Morrill Act of 1890, mandating that all states had to either provide separate educational facilities for blacks or admit them to existing colleges. All southern and border states opted to establish “separate but equal” agricultural and industrial schools for blacks in order to get federal money for white land-grant colleges. These facilities were never equal, and consequently, public HBCUs have never come close to their white counterparts financially or academically [9],[11].

3 Krieger et al. pointed out that the COVID-19 Racial Data Tracker reported 6,448, 573 cases as of September 13, 2020, of which 37.5% were missing data on race [18]. Table 1 also shows that race is unknown for 33.3% of COVID-19 cases, 6.5% of COVID-19 deaths, and 7.3% of hospitalizations.

4 Although overt racial discrimination in housing is illegal, many covert *de facto* methods continue to exist, which result in racially segregated neighborhoods. Studies show that poor Black neighborhoods tend to lack parks or other greenspaces, dedicated trails for walking or biking, access to healthy food, clinics, or properly maintained infrastructure, amenities, or appliances. On the contrary, they are frequently characterized by high crime, noise and air pollution, etc. and often located within the close proximity of toxic waste sites, factories, highways and other places of health hazard [21].

5 Syndemic perspective or approach involves the consideration of biological connections (i.e., disease concentrations and disease interactions) under the prevailing social structures and social relationships, e.g., social inequality and injustice) as well as socio-genic environmental conditions, e.g., hazards of the built environment, toxic commodities, pollution, etc. Stated differently, syndemics develop under health disparity, caused by poverty, stress, structural racism, pollution, etc. Unlike comorbidity, which focuses on disease co-occurrence with or without interaction, syndemic theory stresses on adverse disease interactions (i.e., how the presence of one disease or disorder enhances the consequences of other diseases or disorders), their causes (social environments including social structures and social relationships), and consequences for human life (increased risks of exposure, morbidity, and mortality) [22].

6 While these responses were given by interviewees and found in some news articles [24],[25], other explanations could be that enrollments at HBCUs are generally lower, testing may not be widely available at points [26], testing programs on campus may have been delayed, or some campuses may have been closed [27] to avoid higher infections.

7 A survey conducted by the National Foundation for Infectious Diseases (NFID) between December 10–21, 2020 revealed a significant gap in vaccine confidence among Black adults. Fewer than one-quarter of respondents responded ‘extremely’ or ‘very’ confident in COVID-19 vaccine efficacy, safety, or adequate testing among Black adults; only 16% were reportedly confident that the vaccines would be distributed equitably [52]. Even in an earlier survey conducted between November 18–29, 2020 by Pew Research Center, only 42% of Black American adults were inclined to get vaccinated when available, compared to 63% of Hispanic and 61% of White Americans [53]. Sadly, at least to some, the distrust in the U.S. public health service among African Americans is deeply rooted in the historical exploitation and abuse of Black bodies in the name of medical progress (e.g. Tuskegee experiment) to justify lower standard of healthcare and other forms of scientific racism [54].
